# Answers to Questions on the Teeth of the White, Mixed and Black Races

**Published:** 1882-11

**Authors:** 


### ARTICLE III.

### *Answers to Questions on the Teeth of the White, Mixed and Black Races.*

---

Professor W. H. Morgan, of Vanderbilt Dental College, says :

"All that relates to the negro, either in a moral, or political point of view, has been greatly intensified in interest to the philanthropist, because of his altered relation to the people of this entire country, but especially to the whites of the South. And while he is, perhaps, for a great while to come, not to be a very important factor in the practice of dentistry, anything relating to his physical well being must interest the earnest seeker after truth who may belong to our calling. When I entered the dental profession, now nearly forty years ago, the opinion prevailed among dentists and physicians that the colored population of the slave states entire, were blessed with much better teeth than the whites of the same region. A little observation showed the fallacy of this opinion, that in fact their teeth were in quality far below those of the whites. A little investigation showed that this opinion resulted from superficial knowledge, that the careless observer was misled first, by the glaring contrast between the color of the teeth and gums, for it must be borne in mind that the pigment giving color to the skin does in a large majority of cases also color the lips and gums.

"Then it is true that the negroes' front teeth do not, under like circumstances, decay near so early or certainly as in the white races, mainly because the dental arches or maxillary bones are almost universally much larger proportionately than in the white races. Under similar condition the negro and all who have an admixture of his blood, lose their teeth by decay, at a much earlier age than the whites. You ask me the cause of this, I answer, first, the inferior texture or quality of his teeth, and this fact applies to all the tissues of his body. He is, without exception, of scrofulous diathesis, all his tissues are softer or more loose in texture than the whites. True his bones are larger, but they are less dense in their structure, and being softer are less liable to fracture. Their muscles are larger, but are less solid, he consequently wastes more rapidily when diseased. Destructive inflammation occurs more readily, and to a greater extent from like causes than in the whites. He succumbs sooner to inflammatory disease than the white races in the same country, and laboring under the same disease. In one notable exception he is exempt, (yellow fever), in all others he is more liable to disease. Lessions occur at an earlier date and terminate fatally more frequently, consequently he is shorter lived. So marked is this tendency to destructive inflammation that it is almost useless to attempt to save pulpless teeth, except those which have single roots. It is time and labor nearly thrown away if expended on the bicuspids or molars, after the devitalization of the pulp, only a short time elapsing after filling, before inflammation and abscess occurs, save in a few exceptional cases. To sum up the negro is scrofulous, his teeth are large, of soft texture, the bicuspids being deeply marked with fissures, often to the extent of exposure of the dentine, they are subject to early decay and loss. When the pulp is lost the roots are a much greater source of irritation than in the whites; the gums take on fungous growths much more readily, and he loses his teeth, especially those posterior to the canines, at a much earlier age. He succumbs more easily to disease, and on an average, I believe, does not live to so great an age. The mulatto universally has poor teeth; they are more frequently of a pearly cast than the pure black and decay in early life. In a practice of thirty-five years in the States of Kentucky and Tennessee, I have seen but two mulattoes of mature age who had sound teeth. If the mulatto co-habits with the white, their progeny, to a large extent die before maturity, and physical degeneracy is marked and rapid. If cohabitation is with the mulatto the family is usually small and short lived, if with the black their issue is an improvement on the mulatto, the teeth always partaking of the general physical characteristics."

J. H. Coyle, D. D. S., Thomasville, Georgia, says: "The teeth of the pure blooded negro are very much better than those of the white race. The teeth of the mixed race are not as good as those of the negro. They are not as good as those of the white. Ninety per cent. of the irregularities exist in the white race and ten per cent. in the mixed. I never saw a case of irregularity in the pure blooded negro. The nearer you approach the normal status, that is a full white, there is improvement in the general character of the teeth.

"The gums of the negro are always in the very highest state of health; variable in the white; and the only condition I have ever noticed as occurring frequently in the gums of the mixed race, is more frequently a spongy condition.

"The pure blooded negro taken from the plantation and converted into a house servant, partaking of the same food as the white, rapidly lose their teeth by caries, especially the next generation of them raised up under such surroundings."

Dr. T. C. Edwards, of Brownsville, Tennessee, says:

"The teeth of the negro are not as good as those of the white race, in this comminity at least.

"The teeth of the mixed race are not as good as those of the black, therefore not as good as those of the white race. There is but little irregularity of the teeth among the negroes.

"I do not see that a predominance of "white blood" makes any material difference unless for the worse. I never believed that the mixed race had any permanence. I believe they will eventually go back either to the one or the other type, or degenerate and become extinct.

"The negro does not have gums of as delicate structure as the white race, but they yield readily to disease, (as do the teeth of the negro; when caries begins in their teeth they seem to melt away as wax.)

"The gums of the negro as well as the mixed race, as a rule, are not healthy, owing to their careless and filthy habits, and disregard of health rules. This may explain in a great measure the condition of their teeth."

Dr. J. P. Johnston, of Marion, Alabama, says."

"There must be some standard by which to judge. I will base my opinion, therefore, on the ground that the teeth of both races received the same attention. As a rule the teeth of the negro are much larger and more fully developed, result of mode of life, diet, etc. But to answer the questions: The teeth of the negro are better than the white race. The teeth of mixed race are not as good as those of the black. They are as good as those of the white race.

"Irregularities of the negro's teeth are very rare; largely predominates in the white race over the mixed or black. As the blood of the white race predominates the teeth are softer, enamel not so perfect.

"The gums of the negro and mixed races are better than the white race."

Dr. E. M. Allen, Marietta, Georgia, says:

"I think the teeth of the negro better than the white race. I do not think the teeth of the mixed race as good as those of the black. They are probaly as good as the teeth of the white race unless they inherit from the blood of the white greater tendency to decay. Much less number of irregularities occur in the black than white race.

"There is a perceptible change in the teeth as the blood of the white predominates."

Dr. W. D. Dunlap, Selma, Alabama, says:

"The teeth of the negro race in this region, whether in town or country, are inferior to the teeth of the white race. The mixed race have more defective teeth than either the black or white, more sensitive and difficult to preserve.

"Irregularities largely predominate in the white race."

Dr. A. F. Claywell, Lebanon, Tennessee, says:

"Under similar conditions of habit and occupation, there is no appreciable difference in the teeth of the races.

"The teeth of the mixed race are far inferior to those of either the black or white races. As the blood of the white race predominates irregularity increases. The gums of the white race are inferior to those of the mixed or black race, black best."

Dr. S. P. Buatt, Bastrop, Louisianna, says:

From theory and experimental facts, negro as an inferior being in existence and purpose, practically proves his inferiority to all others in his dental development. In proportion to their proximity to the white race their teeth are superior to the blacks.

"Their teeth are not as good as those of the white race. In their respective order as primitive race classes, postulated as perfect physical developments, they are equally normal. But as irregularity is an abnormal condition, the proportion is with the whites, as the most advance in civilzation, and their approximate races.

"As the blood of the white race predominates, the superiority exists.

"In normal subjects there is no discrepancy in comparison, but as diseased conditions are sequences of vice and abnormalities, it is a compendium of physical depravities peculiar to hygienic aberations, consequently holds its comparison within the range of vital degradation, and cannot be estimated by observation aside from their concomitant causes."

As will be observed there is quite a diversity of opinion as to some of the questions. Whether latitude, longitude or altitude, has anything to do with it we are not able to say. One question all agree on, and that is, the regularity of the negros' teeth. From this we are to learn that nature, left undisturbed, will build out in symmetry and form beautiful to behold. The most perfectly developed beings ot this continent are the negroes. Take him in his natural state from under the influences of modern civilization, he presents the most symmetrical form of all races. With civilization the negro degenerates rapidly, as is shown from the above answers.

Another question nearly all seem to agree upon, is the injurious effect of the amalgamation of the races. The whole physical structure of the mulatto is inferior to either the white or black race. They are very geneally of a nervvous temperament, often in combination with the lymphatic. Their intellect is much better, and capacity for learning much greater than the pure negro. We believe it is generally conceded that the negro is the best natural singer of any nation on earth. We are not prepared to say about the native African. Cannot very much of this be attributed to their perfect physical development, especially the perfect oral development of the races? It is a fact that the mixed race, as they approximate the white race, lose this heaven born gift in its natural purity and volume. We leave the above question open to the profession. There is much to to be learned from comparison and observation. And with this subject fully developed light will be thrown in dark places.—*Southern Dental Journal.*

---

